# Present Status and Future Challenges of New Therapeutic Targets in Preclinical Models of Stroke in Aged Animals with/without Comorbidities

**DOI:** 10.3390/ijms19020356

**Published:** 2018-01-25

**Authors:** Aurel Popa-Wagner, Daniela-Gabriela Glavan, Andrei Olaru, Denissa-Greta Olaru, Otilia Margaritescu, Oana Tica, Roxana Surugiu, Raluca Elena Sandu

**Affiliations:** 1 Griffith University School of Medicine, Gold Coast Campus, QLD, Queensland Eye Institute, Brisbane, QLD 4101, Australia; 2 Department of Functional Sciences, Center of Clinical and Experimental Medicine, University of Medicine and Pharmacy of Craiova, 200349 Craiova, Romania; roxana.surugiu07@gmail.com (R.S.); ralucasandu80@gmail.com (R.E.S.); 3 Psychiatry Clinic Hospital, University of Medicine and Pharmacy of Craiova, Petru Rares Street 2, 200349 Craiova, Romania; Danaglavan@gmail.com; 4 Department of Ophthalmology, University of Medicine and Pharmacy of Craiova, 200349 Craiova, Romania; andrei8317@gmail.com; 5 Medlife Clinic, Department of Ophthalmology, 200349 Craiova, Romania; denissagretaolaru@gmail.com; 6 Department of Neurosurgery, University of Medicine and Pharmacy of Craiova, 200349 Craiova, Romania; omargaritescu1970@gmail.com; 7 Department of “Mother and Child”, University of Medicine and Pharmacy of Craiova, 200349 Craiova, Romania; oana.banica@umfcv.ro

**Keywords:** aging, stroke, regeneration, rehabilitation, therapy, animal models

## Abstract

The aging process, comorbidities, and age-associated diseases are closely dependent on each other. Cerebral ischemia impacts a wide range of systems in an age-dependent manner. However, the aging process has many facets which are influenced by the genetic background and epigenetic or environmental factors, which can explain why some people age differently than others. Therefore, there is an urgent need to identify age-related changes in body functions or structures that increase the risk for stroke and which are associated with a poor outcome. Multimodal imaging, electrophysiology, cell biology, proteomics, and transcriptomics, offer a useful approach to link structural and functional changes in the aging brain, with or without comorbidities, to post-stroke rehabilitation. This can help us to improve our knowledge about senescence firstly, and in this context, aids in elucidating the pathophysiology of age-related diseases that allows us to develop therapeutic strategies or prevent diseases. These processes, including potential therapeutical interventions, need to be studied first in relevant preclinical models using aged animals, with and without comorbidities. Therefore, preclinical research on ischemic stroke should consider age as the most important risk factor for cerebral ischemia. Furthermore, the identification of effective therapeutic strategies, corroborated with successful translational studies, will have a dramatic impact on the lives of millions of people with cerebrovascular diseases.

## 1. Age as an Important Factor in Preclinical Studies of Stroke

Age is the most important risk factor for cerebral ischemia and recovery after cerebral ischemia. A large spectrum of factors, like genetic, epigenetic, or environmental factors, contribute to the aging phenotype. Organismal senescence affects, first of all, the cardiovascular system and the immune system [1]. Other changes include a decline in sensory function, memory, and cognition [2,3]. Therefore, identifying key factors contributing to aging will improve our understanding of the aging process. Studies conducted on aged (2-year-old) rats demonstrated that there was more severe impairment of behavioral outcomes and poorer neurological functional recovery after ischemia in older rats than in younger animals [4,5,6,7,8,9,10,11,12]. Having in mind that epidemiological studies revealed that human stroke occurs more often in late middle age (50–70 years) than at older ages (more than 70 years) it comes as quite a surprise that young animals are mostly used to test new drugs for stroke. By now, however, there is a number of reports on age-related alterations in the response to stroke at transcriptional, cellular, and electrophysiological level [11,13,14,15,16,17,18]. Therefore, extrapolating results from young animals to aged humans could lead to erroneous conclusions [19,20].

The aged rodent model offers a useful tool to investigate mechanisms and treatments of ischemic stroke in preclinical studies. However, the models in aged animals have to be designed to (i) create a reproducible lesion which mimics the human pathophysiological changes; (ii) be minimally invasive, and (iii) allow objective measurement and analysis of tissue damage after cerebral ischemia. In agreement with this concept, previous studies have shown that mortality in post-stroke aged rats is higher compared with young animals, most likely because the lesion appears on a background already altered by senescence itself. 

At the physiological level, functional and cognitive decline are closely connected to morphological changes of the brain during the aging process. 

Imaging techniques Positron Emission Tomography (PET or Magnetic Resonance Imaging (MRI) have revealed a significant reduction in the cerebral blood flow (CBF), mostly in the cortex, which may be linked to the morphological changes in the aged brain. Overall, cerebrovascular dysfunction associated with metabolic changes due to senescence increases the vulnerability of brain to ischemic-hypoxic injuries like stroke. Cerebral ischemia occurs frequently in the elderly, and increases the vulnerability of the aged brain, leading to unfavorable recovery of physical and cognitive functions. Although imaging techniques have already been used in numerous studies in animal models of stroke [21], few groups have applied MRI methods to characterize and monitor the dynamics of ischemic lesions in aged ischemic animals [22,23,24,25]. The aged brain displays a higher susceptibility to hypoxia compared with young animals in the acute phase of stroke [25,26]. On MRI images, aged ischemic rats displayed more severe lesions, which had similar localizations, but higher incidence and more rapid appearance than in the young rats [22,23,27,28]. With the use of functional Magnetic Resonance Imaging (fMRI), it was demonstrated that patterns of bihemispheric reorganization (increase of the fMRI response in the ipsilateral somatosensory cortex and bilateral thalamic activation) after permanent middle cerebral artery occlusion (MCAO) in aged rats, were the same as in young animals, although the overall time course of recovery in aged rats was more prolonged than that in young rats [24,29]. 

Studies using electrophysiological techniques, and in particular, electroencephalography (EEG), in ischemic aged animals are quite a few. EEG has been used as a tool for verifying the success of the occlusion [22], for identifying the effect of hypothermia on neuronal functions [30], or for evaluation of neurological and electroencephalographic asymmetry after unilateral MCAO [31]. Aged rats exhibited much more asymmetry in cortical EEG between both hemispheres, which coincided with the more severe unilateral somatosensory deficits in aged than in young rats [32]. 

Cortical spreading depression (CSD) and periinfarct depolarization (PID) occur spontaneously in various experimental models of stroke, and the brain after stroke, causing secondary neuronal damage and infarct expansion [33]. Recently, it was found that there was an inverse neurovascular coupling with SD in the old, but not in the young animals, suggesting that (mal)adaptation of cerebrovascular function with aging impairs the SD-related cerebral blood flow (CBF) response [34]. In the clinic, spreading depolarizations also occur in patients with ischemic stroke [35].

Inflammation is an important promoter of atherosclerosis and plaque instability, both associated with an increased risk of stroke in the elderly. Microglia cells, the resident macrophages in the brain, are highly dynamic cells that regulate homeostasis in physiological conditions, and constantly palpate the surface of neurons for microlesions with motile processes [36,37]. Thus, subtoxic insults, such as inflammation, may cause the exposure of the “eat-me” signal phosphatidylserine (PS) on the surface of stressed but viable neurons. Migrating microglia detect the exposed “eat-me” signals and engulf­ment of neurons or parts of neurons exposing such signals, follows. This process has been coined primary phagocytosis or “phagoptosis” [38,39]. However, toxic neuronal insults, such as dying neurons after stroke, irreversibly expose the “eat-me” signal recognized by primed microglia, resulting in phagocytosis of the dead neurons, or so-called secondary phagocytosis [40]. There are “eat-me” signals other than phosphatidylserine. The complement components C1q and C3, which are produced by microglia and astrocytes, may induce phagocytosis by binding opsonized/altered neuronal surfaces. In this process C1q promotes the conversion of C3, expressed by microglia, to C3b. C3b then opsonizes neurons and is recognized through complement receptor 3 (CR3) expressed by activated microglia.

Following an acute injury such as stroke, the apoptotic neurons release a chemotactic signal, such as fractalkine/CX3CL1, [41,42], and microglia expressing the fractalkine receptor (CX3CR1), promote phagocytosis of apoptotic cells expressing CX3CL1 [42]. The aging brain reacts strongly to ischemia-reperfusion injury with an early inflammatory response [26,43]. This inflammatory response is characterized by increased chemokine expression (CCL2, CXCL1, CCBP2), cytokine expression (Tumor Necrosis Factor alpha, TNFα; Interleukin 1, IL1; Interleukin 6, IL6), and increased cell death. Recently, Liu and colleagues [44] identified 255 aberrantly expressed long noncoding RNAs that were mainly associated with inflammatory pathways in the ischemic mice. 

Clinical studies in humans have revealed a peripheral increase of a subset of the T-cell population within 48 hours after an acute ischemic cerebral event. More specifically, Tuttolomondo and colleagues found a higher peripheral frequency of CD28 null cells in subjects with acute ischemic stroke, as compared to controls, and suggested a possible role for a T-cell component also in ischemic stroke setting [45,46].

At transcriptional level, five upregulated inflammation-related genes in the peri-infarcted region (*Ptpr*c, *Ptges3*, *Tgfbr1*, *IL6*, *Rps2*) were found in aged rats, but not in young animals [47]. At the molecular level, *Tgfr1* gene was shown to be overexpressed in aged rats only. This gene, which is closely related to increased inflammation in the aged brain after stroke, promotes increased fibrosis and scar formation [26,47]. Cytokine-mediated alteration of neuronal membrane and axonal conduction may contribute to increased neuronal deficit in the aged ischemic brain after stroke [48]. Overall, these pro-inflammatory reactions cause an increased blood brain barrier (BBB) permeability and reduced functional recovery, and promote early scar formation and increased astrogliosis associated with tissue fibrosis [43,49]. 

Besides inflammation, oxidative stress is another central player in brain aging, which affects, in a negative mode, the capacity of aged cells to counteract the neurotoxic consequences of an ischemic lesion. At the molecular level, some mediators of senescence, such as oxidative stress or epigenetic factors like DNA damage (by methylation or acetylation), aberrant somatic mutation, and shortage of telomeres, may be identified. In addition, some proteins changes are involved in age-associated degenerative phenomena, such as post-translational modification, misfolding, and aberrant aggregation [50]. 

Oxidative stress is part of many age-related diseases, including stroke. The brain is the organ with the highest level of oxidative metabolism, and mitochondria are the main player in the energetic pathway through electron carrier complexes in the respiratory chain, but also in other important processes, like fatty acid oxidation, reactive oxygen species (ROS) production, and apoptosis. For many years, large amounts of data on the possible contribution of ROS to the mechanisms of aging has been published. Much evidence supports the idea of a powerful association between longevity and antioxidant capacity of cells. On the other hand, some studies reported that transgenic animals, which lack key components of the antioxidant defense, do not show clearly shorter lifespan [51]. Likewise, Desler and colleagues showed that accelerated development of aging phenotypes, through mitochondrial DNA (mtDNA) mutations, was not correlated with increased ROS, and that the process involved increased apoptosis by early activation of caspase 3 [52]. However, whether it is a primary cause, or a secondary effect, is still an open question. Further, epigenetic events caused by ischemic stroke, including DNA methylation and changes in the state of histone acetylation, or microRNAs, may impact on the redox state in neurons, glia, and endothelial cells of vascular neural network [53].

Previous studies showed that mitochondrial dysfunction occurring during the normal aging process predisposes to increased oxidative damage after brain injury [54]. Buga and colleagues [47,55] demonstrated that at transcriptional level, the antioxidative capacity of aged animals was decreased compared to young ones. Genes involved in the antioxidative defense capacity, such as superoxide dismutase 2 (SOD2), were downregulated in the aged brain. Another gene, catalase (CAT) was unchanged in response to injury in the aged brain [47]. Decreased cellular antioxidant defense capacity, together with an early inflammatory reaction, may contribute to poor prognosis during the recovery phase after stroke in elderly. 

Genetic factors play an important role in the pathogenesis of cerebral ischemia. Recent genome-wide association studies (GWAS) have identified several single nucleotide polymorphisms which have been associated with an increased risk of cerebral ischemia [56,57,58,59,60,61]. Previous studies also suggested that increased DNA damage occurs after cerebral ischemia [62]. This process involves two complementary pathways; active DNA damage mediated by DNA endonuclease, and passive DNA damage induced by oxidative stress, which is endonuclease-independent [63]. Passive DNA damage is an important factor during the reperfusion period, which affects brain tissue at many levels (neuronal and astrocyte viability, endothelial cell integrity). In this case, DNA repair capacity of brain tissue becomes vital to limit ischemic consequences. The principal DNA repair pathway is the base excision repair pathway (BER) that fixes DNA degradation after injuries, and promotes genomic integrity [63]. Some evidence suggests that the efficacy of the BER pathway is decreased in the aged brain, and this may at least partly explain the increased vulnerability after lesion in aged animals [64]. 

Experimental evidence suggests that the severity of injury during cerebral ischemia is significantly influenced by age, not only at structural and functional levels, but also at transcriptional levels. These age-related changes in the transcriptional activity of brain are associated with an increased vulnerability and reduced functional recovery after focal cerebral ischemia [47]. Increased expression of genes involved in DNA damage (*Gadd45a, Hus1, Mdm2, and Tnfrsf7*), and decreased expression of anti-apoptotic genes (*Traf1, Traf4, Trp53*) or genes involved in neuroprotection after injuries (*Fgf22, Ngf, Olig1*), are part of the genetic profile of aging [47]. In agreement with this, mutations in some genes involved in DNA repair and maintenance, like *Ercc*2 or *Wrn*, accelerate the aging process [65,66]. 

During the recovery phase of stroke, crucial processes like vasculogenesis, angiogenesis, and neurogenesis, are also strongly influenced by the senescent background. In elderly, there is not only a reduction in cerebral blood flow, but also a reduction in the density of blood vessels, and these changes make the aged brain tissue more vulnerable to hypoxic injuries. Vasoconstriction is known to occur in cerebral arterioles during ischemia [67]. Interestingly, phosphodiesterase-5 (PDE-5) inhibitors, which increase blood flow by inhibition of the enzyme that is involved in maintaining blood vessel tone, have been successfully used in the treatment of hand ischemia [68]. Numerous studies suggest that the vasculature is inflicted by age-specific changes like microvascular degeneration [69]. In this context, current imaging methodologies may play an important role to identify patients at risk. 

Angiogenesis is considered a key factor in post-stroke reorganization processes, but mechanisms that modulate angiogenesis in elderly after stroke are unknown. Gene therapies that target angiogenic factors may be useful for post-stroke treatment and functional recovery. For example, pro-angiogenic factors have been shown to support neurogenesis after stroke, and development of the post-stroke neurovascular niche that could promote neural repair after stroke [70,71,72]. Nevertheless, we have to keep in mind that angiogenesis interacts with the aged brain in different ways, and that many pro-angiogenic pathways that are active after stroke, are also involved in atherosclerotic plaque formation. This process is associated with formation of immature blood vessels that are prone to rupture [73]. In this light, it is crucial to find pro-angiogenic drugs that selectively promote angiogenesis after stroke without favoring atherothrombosis in aged organisms [74,75].

Studies in animal models showed that neurogenesis is impaired in the aged brain [76]. After stroke, in the subventricular zone (SVZ), there is an increased number of newly born neurons, but the ability of these neurons to differentiate into mature neurons is limited in the aged brain [77]. Also, the molecular profiles of repair processes and axonal reorganization after stroke are highly influenced by age, and changes in cortical environment may decrease the repair capacity of the brain after injury [76]. Moreover, the presence of amyloid precursor protein beta (βAPP) and Aβ immunoreactivity in the infarct area indicates that stroke promotes conditions that are favorable to accumulation of neurotoxic factors, such as Aβ, especially in the aged brain, thereby limiting neurogenic events [78]. Quite unexpectedly, middle aged and older mice showed an upregulation of neurogenesis in the contralateral uninjured hemisphere, as opposed to young mice [79]. 

## 2. Comorbidities as Important Factors in Preclinical Studies of Stroke

Healthy aging depends on various factors [80]. The incidence of many diseases increases with advancing age, depending on genetics, biological risk factors, or social factors. These factors influence the progression of functional decline, and promote development of age-associated diseases (cardiovascular disease, cancer, neurodegenerative disorders, etc.). The incidence of metabolic disorders, including obesity, impaired glucose tolerance, and type 2 diabetes, increase with advancing age. All these metabolic changes contribute to the development of age-related cardiovascular and neurodegenerative diseases, and ischemic stroke is one of the most frequent of them [72]. Thus, in elderly, comorbidities like diabetes or arterial hypertension are associated with a higher risk of stroke, increased mortality and disability, and poorer functional status and quality of life. However, most animal models of stroke ignore age and comorbidities frequently associated with senescence, and this could be one of the explanations for unsuccessful bench-to-bedside translation of neuroprotective strategies. Currently, there are several different rodent models that include comorbidities for stroke research, such as spontaneously hypertensive rat models (SHR, SHRP), the streptozocin rat model for diabetes, and high-fat diet or high-sugar diet for Sprague Dawley rats. Studies performed in SHR showed an increased infarct size. Also, by MRI, hyperglycemia was shown to accelerate the infarct progression, especially in cortical areas [81]. But the mechanism of this hyperglycemia-associated infarct progression still remains unclear. Simultaneous presence of two comorbidities, like hypertension and chronic diabetes, significantly increases the level of ischemic damage in animal models, probably due to vascular diabetic complications [20,82]. 

Our knowledge about the molecular and cellular mechanisms underlying this metabolic syndrome, which may include multiple comorbidities, is still poor. Some studies report a strong connection between nutrition, body weight, on the one hand, and increased oxidative stress or pro-inflammatory changes in the brain, on the other hand, which promotes neural imbalance and glucose level elevation. All these factors contribute to the common pathogenic base for metabolic syndrome and cellular stress of the brain [83]. Zhang and colleagues suggested that metabolic inflammatory changes in the brain are connected with the IKKβ/NF-κB signaling pathway [84]. Overnutrition is not only responsible for metabolic inflammation of the brain, but can also induce mitochondrial dysfunction and increased oxidative stress, nicotineamide adenine dinucleotide phosphate (NADPH ) oxidase-dependent, which promotes metabolic syndrome and related diseases [83]. Cellular stress and hyperglycemia are known to accelerate the aging process. In this light, a better understanding of molecular factors and signaling pathways underlying the metabolic syndrome, as well as the contribution of comorbidities to stroke-induced pathological sequelae, may be translated into successful treatments or prevention therapies against age-associated diseases, which would improve lifespan and quality of life. For example, metabolic inflammation can be modulated by rationale nutrition [85].

## 3. Promising New Therapeutic Strategies of Stroke

Treatments against age-related functional deficits or structural changes are limited. Many previous studies have focused on those pathways that are associated with inflammation or oxidative stress after injury. The aged brain has a diminished capacity of regeneration after injuries, and this may be due, in part, to age-related decrease in endogenous neurogenesis and neuroplasticity. Also, age-related changes in brain environment, including immunological or trophic factors, may be detrimental for newborn cell survival or neuronal outgrowth, and may prevent migration of stem cells to the lesioned site. 

Stem cell therapy, which may restore structure and function in diseased CNS, such as after stroke, is a very dynamic field. Two types of stem cell therapy may be distinguished: endogenous and exogenous. A problem usually associated with exogenous stem cell therapy is to find the best way to deliver the cells. Intracranial delivery of stem cells is an invasive method with increased secondary risk, especially in elderly with comorbidities like diabetes or cardiovascular diseases. This method has limitations because an inflammatory response and rejection of the cells might be stimulated. Another method to deliver stem cells is intraarterial administration along with mannitol. Mannitol creates a hyperosmotic environment that is able to disrupt, transiently, the brain–blood barrier (BBB). This disruption increases the permeability of the brain for stem cells. However, this method is associated with an increased risk for hemorrhage or other complications [86]. Potentially safer and more effective therapeutic strategies in this direction attempt to disrupt the BBB and deliver neural stem cells into the brain using MRI-guided focused ultrasound [87]. This method still has potential drawbacks, like autoimmunity reaction. MRI may provide an indispensable non-invasive method for monitoring the regeneration of tissue, identifying potential adverse effects, and establishing the barriers that prevent cell migration at the site of injury [88]. 

Mesenchymal stem cells (MSCs) have been used in the last decade to improve molecular and behavioral indices of recuperation after stroke. Recent studies, using extracellular vesicles derived from mesenchymal stem cells, suggest that this approach is well tolerated, and therefore may offer an additional benefit by eliminating the side effects of stem cell transplantation [89].

It is well known that caloric restriction in aged animals not only extends lifespan and decreases the risk of stroke, but is also associated with less damage and better outcome after stroke [90]. Studies using MRI in aged rhesus monkeys under caloric restriction, have shown that long-term 30% caloric restriction attenuates inflammatory reaction in the aged brain, and prevents weight loss of brain tissue [91]. Aged laboratory Sprague Dawley rats fed ad libitum become obese, and have high insulin levels, and thus represent a “natural” model in which to study the effect of obesity on behavioral recuperation, and the severity of cerebral necrosis after stroke. Recently, Ciobanu et al. [92] showed that in post-stroke, aged, calorie-restricted Sprague Dawley rats, behavioral recuperation is enhanced as compared with ad libitum fed, overweight aged rats. In this setting, there is an early gain in body weight and improved behavioral recovery that requires complex sensorimotor skills, such as the rotating rod and inclined plane tasks, or cutaneous sensitivity and sensorimotor integration or spatial memory that were associated with increased serum glucose, insulin, and IGF1 levels, and with specific changes in gene expression, including downregulation of genes involved in the ubiquitin proteasome degradation system (*Pmds6, Psmc4, Pmc3, Psmb5*), enhanced vasculogenesis (*Ppp2cb*), neuroprotection (*Mapk10*), and reduced apoptosis (*CaMKIIc*). All of these changes have been recorded in parallel with an increased expression of genes that are neuroprotective (*Igfbp3*), support angiogenesis (*Igf2*, *Mapkapk2*), and allow an increase in available energy (*Prkaac/Prkga1*). However, more experimental studies are needed, in order to fully understand the active, complex interrelationships in this pathological setting. 

Among neuroprotective methods, long-term hypothermia has proven to be an efficient method to reduce infarct size and to improve recovery after stroke [72,93,94]. The authors demonstrated, with EEG and MRI techniques, that hypothermia diminished the brain activity at functional (appearance of low-amplitude 4 Hz oscillatory EEG activity similar to those in hibernation-like state) and metabolic levels, and decreased the T2-weighted hyperintense ischemic area by 57%.

Over the last years, nanotechnology has led to the development of different nanoparticle agents for molecular imaging applications or targeted drug delivery into the brain. Nanoparticles not only provide a platform for promising new contrast agents, but also for drug carriers to selectively deliver into tissues [95]. Microparticles of iron oxide are novel contrast agents for molecular MRI, which can target molecules expressed on healthy and diseased endothelial cells, which can aid in detection of new therapeutic targets in vascular diseases like stroke [96]. This research area is still under development, but offers promising means for direct monitoring of therapeutical intervention in a minimally invasive way [97].

Recently developed genome-editing technologies opened new avenues for stroke prevention by altering genes in adult organisms. Thus, CRISP-Cas9 system, that allows base editing in the genomic DNA, and thus, permanent alteration of genes in adult organisms, can be used in the prevention and treatment of atherosclerosis by correction of atherogenic gene mutations, or insertion of protective genes [98,99].

## 4. Conclusions

**Figure 1:**
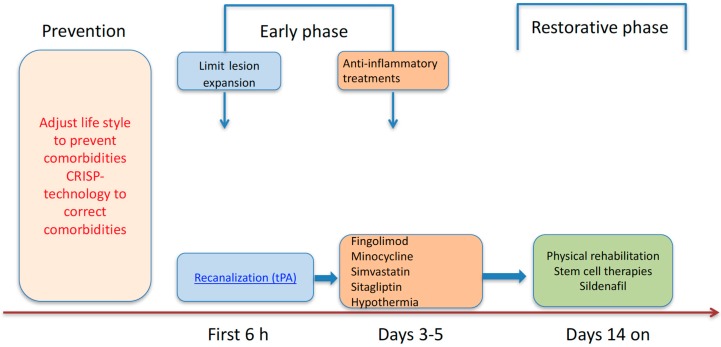
Stage-specific therapeutical approaches in humans and animal models.

Stroke impacts a wide range of systems in an age-dependent manner (Table 1). The aging process has many facets which are influenced by genetic background and epigenetic or environmental factors, which can explain why some people age better than others. The aging process, comorbidities, and age-associated diseases are key factors that are closely dependent on each other. Multimodal imaging offers a useful approach to link structural and functional changes to cerebral ischemia in the aging brain, with or without comorbidities. This can help us to improve our knowledge about senescence firstly, and in this context, aid in elucidating the pathophysiology of age-related diseases and development of prevention or therapeutic strategies. These processes, including potential therapeutical interventions for cerebral ischemia, need to be studied first in relevant preclinical models using aged animals with and without comorbidities (Figure 1). The identification of effective therapeutic strategies, which will have a dramatic impact on the lives of millions of people with cerebrovascular diseases, will be the most valuable consequence of successful translational studies.

## Figures and Tables

**Table 1:** Summary of cellular and molecular events after focal ischemia in the aged rat brains as compared to the young post-stroke rat brains.

Event	Main Findings in the Aged Rat Brains	References
Neuroinflammation	Fulminant development of astroglial response; increased BBB permeability	[12,43,49]
Cell death	Accelerated apoptosis; Microglial engulfment of dying neurons	[26,38,39]
Early gene expression	Decreased number of transiently upregulated genes; Early upregulation of genes associated with DNA damage and down-regulation of anti-apoptosis related genes; Increased number of genes associated with phagocytosis; Increased number of genes coding for pro-inflammatory mediators; Persistent upregulation of genes encoding for extracellular matrix degradation	[47,55]
Infarct development	Precipitous development of the infarct	[26,100,101]
Late gene expression	Increased number of downregulated genes Delayed expression of angiogenesis-related genes Increased no of genes involved in fibrosis and scar build-up Disregulation of gene expression for brain development and CNS remodeling	[47,55,72,102]
Angiogenesis	Delayed sprouting angiogenesis and basal lamina build-up, decreased vascular density in the periinfarcted area but increased vascular density beyond the scar region	[72,102]
Neurogenesis	Impaired neurogenesis and decreased expression of the neuronal precursor marker, doublecortin	[71,72,77,79]
Behavioral recovery	Limited behavioral (motor, sensory, working memory) recovery	[26,78,92,103,104,105]

BBB: blood brain barrier; CNS: central nervous system.
